# The product of waist and neck circumference outperforms traditional anthropometric indices in identifying metabolic syndrome in Chinese adults with type 2 diabetes: a cross-sectional study

**DOI:** 10.1186/s13098-021-00653-4

**Published:** 2021-03-26

**Authors:** Yunhong Huang, Liping Gu, Na Li, Fang Fang, Xiaoying Ding, Yufan Wang, Yongde Peng

**Affiliations:** Department of Endocrinology and Metabolism, Shanghai General Hospital, Shanghai Jiao Tong University School of Medicine, 100 Haining Road, Shanghai, 200080 China

**Keywords:** Chinese adults, Metabolic syndrome, Neck circumference, Type 2 diabetes mellitus, Waist circumference

## Abstract

**Background:**

Traditional anthropometric indices are used in diagnosing metabolic syndrome (MetS). This study aimed to propose a novel index, a product of waist and neck circumferences (PWNC), and compared its value with traditional anthropometric parameters in identifying the presence of MetS in Chinese adults with type 2 diabetes mellitus (T2DM).

**Methods:**

From September 2017 to June 2019, a total of 2017 Chinese adults with T2DM from the National Metabolic Management Center were included and categorized into a MetS group (1575 cases) and a non-MetS group (442 cases). Demographic and metabolic characteristics were compared between the two groups, and logistic regression analysis was performed for MetS. Body mass index (BMI), waist-to-hip ratio (WHR), waist circumference (WC), neck circumference (NC) and PWNC were assessed by constructing receiver operating characteristic (ROC) curves, and the area under the ROC curves was compared by DeLong’s test.

**Results:**

Compared with the non-MetS group, men and women with MetS had higher blood pressure; higher levels of fasting plasma glucose, fasting insulin, and triglycerides (TGs); lower levels of high-density lipoprotein cholesterol (HDL-C); elevated homeostasis model assessment of insulin resistance (HOMA-IR); and higher BMI, WHR, WC, NC and PWNC (all *P* < 0.01). Logistic regression showed that PWNC, HDL-C, TGs, HOMA-IR, systolic blood pressure, hypertension and hypotensors were independent risk factors for MetS (all *P* < 0.01). PWNC, WC, NC, WHR and BMI displayed significant values in the ROC for MetS (all *P* < 0.01), while the area under the curve for PWNC was larger than that for traditional anthropometric parameters (WC, WHR and BMI) in both men and women (all *P* < 0.01).

**Conclusion:**

PWNC outperformed traditional anthropometric parameters in identifying the presence of MetS in Chinese adults with T2DM.

## Background

Metabolic syndrome (MetS) is a cluster of interrelated risk factors of metabolic origin such as obesity, hyperglycaemia, dyslipidaemia, and hypertension, which are linked to the development of cardiovascular disease (CVD) and are responsible for the rise in CVD mortality [1]. CVD remains the most common cause of death for adults with type 2 diabetes mellitus (T2DM) [2]. Diabetic patients with MetS have a much higher prevalence of CVD than those without MetS [3, 4]. The MetS definition is useful for physicians to identify patients at high risk for CVD early and reduce the morbidity and mortality of CVD. The diagnostic criteria of MetS vary slightly between guidelines issued by different expert groups [4–10], but all include obesity, which is even identified as a prerequisite for MetS in the International Diabetes Federation (IDF) guidelines [7]. Obesity, particularly central obesity, is associated with insulin resistance (IR) characterized by hyperinsulinaemia and hyperglycaemia, leading to hypertension and dyslipidaemia and promoting atherosclerotic cardiovascular diseases. Obesity is the central component of MetS.

Obesity can be assessed by numerous methods. Computed tomography, magnetic resonance imaging, and dual-energy X-ray absorptiometry can precisely quantify body fat and fat distribution, but they are costly and sophisticated and cannot be applied in routine clinical practice. Instead, anthropometric measurements are considered simple, quick, inexpensive, and practical methods that are internationally accepted and clinically used in diagnosing MetS. Three traditional anthropometric parameters, body mass index (BMI), waist circumference (WC), and waist-to-hip ratio (WHR), have been included in diagnostic criteria since 1998, when the World Health Organization (WHO) proposed the first MetS definition [5]. BMI, used in definitions of WHO [5] and American College of Endocrinology (ACE) [8], well reflects fat quantity but not fat distribution and may omit visceral fat deposits. However, WC is a surrogate marker of abdominal fat accumulation; it is widely accepted and used as a diagnostic criterion of MetS by many organizations, including the European Group for the Study of Insulin Resistance, the Adult Treatment Panel III of the National Cholesterol Education Program (NCEP ATP III) [6], the American Heart Association/National Heart, Lung, and Blood Institute (AHA/NHLBI) [9], and the ACE [8], and even became a necessity in the IDF definition in 2005 [7]. In 2009, the IDF, along with several organizations (including the AHA, the NHLBI, the World Heart Federation, the International Association for the Study of Obesity, and the International Atherosclerosis Society), harmonized the criteria defining MetS and changed WC from diagnostic requirements to one of the criteria [10]. WHR, the ratio of WC and hip circumference (HC), is only used in the WHO definition; it considers both abdominal obesity and lower-body fat deposits that are distributed subcutaneously over the hips and buttocks.

Unlike HC, an indicator of lower-body subcutaneous fat that plays a protective role against MetS, neck circumference (NC), a marker for upper-body subcutaneous fat, is associated with cardiometabolic risk and contributes to determining MetS risk beyond classical anthropometric indices [11]. In persons with upper-body obesity, the amount of subcutaneous fat typically exceeds visceral fat by twofold or threefold [12]. NC may confer additional risk beyond visceral obesity. It has the same power as WC for identifying metabolic disorders in a Chinese population [13]. These results raise the question of whether WC and NC considered together by calculating their product will reveal that the product of WC and NC (PWNC) is superior to other anthropometric indices for identifying MetS. Therefore, this study explores whether PWNC can outperform traditional anthropometric indicators for MetS in type 2 diabetic adults.

## Materials and methods

### Study subjects

The present study was a cross-sectional study comprising 2017 diabetic subjects recruited from the National Metabolic Disease Management Center (MMC) in Shanghai General Hospital from September 2017 to June 2019. Participants were included in the study if they were (i) diagnosed with type 2 diabetes mellitus (T2DM) according to the guideline [5] proposed by the World Health Organization in 1999 and (ii) Chinese adults aged 30 years and above. On the other hand, subjects were excluded if they (i) had major medical conditions such as liver and kidney dysfunction, severe heart failure and neurological diseases; (ii) had goitre and thyroid dysfunction; (iii) had cervical spine abnormalities or Cushing syndrome; or (iv) did not have complete clinical data. All subjects provided informed consent prior to their inclusion, and the research was carried out in compliance with the Declaration of Helsinki. The study protocol was approved by the ethics committee of the Shanghai General Hospital.

### Study design

MetS was defined using the IDF definition (revised in 2009) [10] and could be diagnosed when any three or more of the following five conditions were fulfilled: ① elevated waist circumference with ethnic-specific cut point, defined as waist circumference ≥ 90 cm in men and ≥ 80 cm in women (as recommended for the Asian population based on WHO recommendations); ② serum triglycerides ≥ 1.7 mmol/L or drug treatment for elevated triglycerides; ③ serum high-density lipoprotein (HDL) cholesterol < 1.0 mmol/L in men and < 1.3 mmol/L in women or drug treatment for low HDL cholesterol (use one or more of fibrates or niacin); ④ blood pressure ≥ 130/85 mmHg or using hypotensors (antihypertensive drugs); and  ⑤ fasting plasma glucose (FPG) ≥ 100 mg/dL (5.6 mmol/L) or drug treatment for elevated blood glucose.

Participants’ anthropometric parameters were taken by two trained nurses. Weight and height were measured with a height and weight meter (OMRON HNH-318; OMRON Corporation, Kyoto, Japan). WC was measured at the horizontal plane of the midpoint between the inferior costal margin and the superior border of the iliac crest, with the subject standing upright and wearing thin clothing. Hip circumference was measured at the level of the widest portion of the buttocks. NC was measured at the seventh cervical margin and below the laryngeal prominence (Adam’s apple) with the subject sitting upright and face directed forward. WC, NC, and hip circumferences were measured to the nearest 0.1 cm using a non-elastic tape. BMI was calculated by dividing the subjects’ weights by the square of their heights (kg/m^2^). WHR was the ratio of waist-to-hip circumference. PWNC was calculated by the product of WC (cm) and NC (cm).

Blood samples were collected after a 12 h overnight fast. Plasma glucose, total cholesterol, high density lipoprotein, triglycerides, and other biochemical indicators were determined using an auto analyser (Hitachi 7600, Hiratsuka, Japan) with WOKO reagent (Sanwa International Co., Hiratsuka, Japan). HbA1c was detected by high-pressure liquid chromatography. Fasting serum insulin (FINS) was determined by immunochemiluminescence (Abbott, Chicago, USA). Homeostasis model assessment of insulin resistance (HOMA-IR) was calculated by HOMA-IR = FPG*FINS/22.5.

### Statistical analysis

Data are presented as the mean ± standard deviation for normally distributed variables, median with interquartile range (25th–75th percentile) for skewed data, and percentages for categorical variables. Intergroup comparisons were conducted using unpaired Student’s t-tests for normally distributed data or the Mann–Whitney U-test for skewed data. Binary logistic regression analysis of the variables was performed to identify risk factors for the presence of MetS, and the results were expressed as odds ratios (ORs) with 95% confidence intervals (CIs). The indicative values of anthropometric parameters for the presence of MetS were calculated by constructing receiver operating characteristic (ROC) curves, and DeLong’s nonparametric approach [14] was used to compare the areas under the ROC curves. Youden’s index was applied to identify the optimal cut-off point for the indicator. All *P-*values were 2-sided, and the results were considered statistically significant if the *P*-value was < 0.05. Statistical analyses were carried out using SPSS statistical software for Windows (version 20.0, IBM Corp., Armonk, NY, USA).

## Results

### Demographic and clinical characteristics according to the presence or absence of MetS

Of the 2017 subjects, 1575 (78.1%) were diagnosed with MetS; 1038 (77.7%) were men, and in women, 537 (78.9%) were MetS (Table 1). Compared with the non-MetS group, subjects in the MetS group had higher systolic and diastolic blood pressure (total: *P* < 0.01, men and women: *P* < 0.05), higher levels of FPG (men and total: *P* < 0.01, women: *P* < 0.05), FINS (all *P* < 0.01), HOMA-IR (all *P* < 0.01), and TGs (all *P* < 0.01), had lower level of HDL-C (all *P* < 0.01). Total cholesterol (TC) levels were elevated in men (*P* < 0.01) but not in women (*P* > 0.05), and 2hPG levels were elevated in women (*P* < 0.05) but not in men (*P* > 0.05). All anthropometric parameters for obesity, including traditional parameters (BMI, WC and WHR) and novel parameters (PWNC and NC), were higher (all *P* < 0.01) in the MetS group than in the non-MetS group in both sexes. There were no significant differences in age, diabetes duration, HbA1c or LDL-C between the two groups (all *P* > 0.05) (Table 1).

**Table 1:** Demographic and clinical characteristics according to the presence or absence of MetS

Characteristics	Total (n = 2017)		Male (n = 1336)		Female (n = 681)	
	MetS (n = 1575)	Non-MetS (n = 442)	MetS (n = 1038)	Non-MetS (n = 298)	MetS (n = 537)	Non-MetS (n = 144)
Age (year)	50.2 ± 11.7	51.5 ± 10.7	48.5 ± 11.5	50.8 ± 10.8	53.3 ± 11.5	53.0 ± 10.6
Duration (year)	0.6 (0.1–6.9)	1.5 (0.1–8.2)	0.4 (0.1–6.2)	1.2 (0.1–7.6)	1.4 (0.1–8.4)	1.9 (0.2–9.9)
PWNC (cm^2^)	3862 ± 481**	3065 ± 405	3996 ± 445**	3221 ± 347	3603 ± 440**	2741 ± 314
BMI (kg/m^2^)	26.8 ± 3.5**	22.6 ± 2.5	27.0 ± 3.3**	22.8 ± 2.4	26.5 ± 3.7**	22.0 ± 2.5
WC (cm)	95.3 ± 6.9**	84.3 ± 6.5	96.2 ± 6.7**	85.5 ± 6.1	93.6 ± 7.0**	81.9 ± 6.4
NC (cm)	40.5 ± 2.9**	36.2 ± 3.0	41.5 ± 2.4**	37.6 ± 2.4	38.4 ± 2.6**	33.4 ± 2.1
WHR	0.97 ± 0.04**	0.92 ± 0.05	0.97 ± 0.03**	0.92 ± 0.05	0.96 ± 0.04**	0.91 ± 0.06
SBP (mmHg)	132 ± 17**	121 ± 14	132 ± 16*	121 ± 16	134 ± 18**	120 ± 12
DBP (mmHg)	79 ± 10**	73 ± 9	80 ± 10*	74 ± 9	77 ± 10*	71 ± 8
HbA1c (%)	8.3 (7.1–10.0)	8.1 (6.8–10.6)	8.4 (7.0–10.1)	8.3 (6.8–10.8)	8.1 (7.1–9.8)	8.0 (6.9–9.9)
FPG (mmol/L)	7.8 (6.5–9.6)**	7.0 (5.9–8.9)	7.8 (6.5–9.6)**	7.0 (5.9–8.7)	7.8 (6.5–9.5)*	7.1 (5.9–9.3)
2hPG (mmol/L)	13.9 (10.9–17.2)*	13.1 (10.1–17.5)	13.7 (10.7–17.0)	13.2 (10.2–17.5)	14.0 (11.2–17.1)*	12.9 (9.7–17.8)
FINS (pmol/L)	69 (50–93)**	32 (21–44)	69 (50–91)**	31 (21–44)	68 (48–95)**	32 (21–45)
HOMA-IR	4.09 (3.03–5.70)**	1.69 (1.18–2.25)	4.05 (3.05–5.62)**	1.76 (1.19–2.21)	4.18 (2.99–6.11)**	1.62 (1.13–2.33)
TC (mmol/L)	4.83 ± 1.30*	4.67 ± 1.10	4.80 ± 1.31**	4.56 ± 1.10	4.89 ± 1.28	4.90 ± 1.06
TG (mmol/L)	1.86 (1.31–2.64)**	1.13 (0.88–1.43)	1.92 (1.36–2.79)**	1.12 (0.85–1.42)	1.72 (1.23–2.41)**	1.15 (0.90–1.45)
HDL-C (mmol/L)	0.94 (0.82–1.11)**	1.13 (1.05–1.33)	0.90 (0.79–1.03)**	1.10 (0.99–1.25)	1.04 (0.90–1.22)**	1.24 (1.09–1.43)
LDL-C (mmol/L)	2.87 ± 0.91	2.75 ± 0.92	2.85 ± 0.90	2.74 ± 0.94	2.91 ± 0.91	2.78 ± 0.88

Continuous variables are presented as the mean ± 1 standard deviation or median (interquartile range). *MetS* Metabolic syndrome, *BMI* Body mass index, *HbA1c* Glycated haemoglobin A1c, *WC* Waist circumference, *NC* Neck circumference, *PWNC* Product of waist circumference and neck circumference, *WHR* Waist-hip ratio, *SBP* Systolic blood pressure, *DBP* Diastolic blood pressure, *FPG* Fasting plasma glucose, *2hPG* 2-h postprandial glucose, *FINS* Fasting insulin level, *HOMA-IR* Homeostasis model assessment of insulin resistance, *TC* Total cholesterol, *TGs* Triglycerides, *HDL-C* High-density lipoprotein cholesterol, *LDL-C* Low-density lipoprotein cholesterol. For the subjects: *P < 0.05, MetS vs. non-MetS; **P < 0.01, MetS vs. non-MetS

### Logistic regression analysis of MetS risk factors

Binary logistic regression analysis for risk factors for MetS was performed in whole, male and female patients, with the presence of MetS as a dependent variable, the independent variables being age, BMI, WC, WHR, NC, PWNC, systolic and diastolic blood pressure, fasting and 2-h postprandial plasma glucose, HbA1c, FINS, HOMA-IR, TC, TGs, HDL-C, LDL-C, and subjects with hypertension and hypotensors (SHH).

In the whole group, logistic regression analysis showed that PWNC (OR: 1.005, 95% CI: 1.004–1.007), systolic blood pressure (OR: 1.039, 95% CI: 1.017–1.061), SHH (OR: 4.184, 95% CI: 2.299–7.576), HOMA-IR (OR: 4.048, 95% CI: 1.970–8.316), TGs (OR: 4.542, 95% CI: 2.811–7.338), and HDL-C (OR: 0.024, 95% CI: 0.007–0.087) were independent risk factors for MetS (all *P* < 0.01). However, among anthropometric indices, only PWNC was identified as an independent risk factor for MetS; no traditional parameters (WC, WHR, or BMI) entered the equation (Table 2).

**Table 2:** Logistic regression analysis of risk factors for MetS

Risk factors	Total		Male		Female	
	OR	95% CI	OR	95% CI	OR	95% CI
PWNC	1.005**	1.004–1.007	1.030*	1.002–1.058	1.009**	1.005–1.012
SBP	1.039**	1.017–1.061	1.031*	1.003–1.059	1.066**	1.019–1.115
SHH	4.184**	2.299–7.576	2.299*	1.148–4.608	22.73**	5.025–99.99
HOMA-IR	4.048**	1.970–8.316	5.106**	2.113–12.34	1.783	0.433–7.334
TG	4.542**	2.811–7.338	5.179**	2.881–9.308	3.046*	1.047–8.857
HDL-C	0.024**	0.007–0.087	0.041**	0.008–0.217	0.004**	0.000–0.043

*MetS* Metabolic syndrome, *OR* Odds ratio, *95% CI* 95% confidence interval. *PWNC* Product of waist circumference and neck circumference, *SBP* Systolic blood pressure, *SHH* Subjects with hypertension and hypotensors, *HOMA-IR* Homeostasis model assessment of insulin resistance, *TGs* Triglycerides, *HDL-C* High-density lipoprotein cholesterol. For the odds ratio

*P < 0.05; **P < 0.01

In diabetic men, logistic regression analysis also revealed that PWNC (OR: 1.030, 95% CI: 1.002–1.058), systolic blood pressure (OR: 1.031, 95% CI: 1.003–1.059), SHH (OR: 2.299, 95% CI: 1.017–1.061) (all *P* < 0.05), HOMA-IR (OR: 5.106, 95% CI: 2.113–12.34), TGs (OR: 5.179, 95% CI: 2.881–9.308), and HDL-C (OR: 0.041, 95% CI: 0.008–0.217) (all *P* < 0.01) were independent risk factors for MetS. Regarding anthropometric parameters, PWNC, rather than WC, NC, WHR, and BMI, was an independent risk factor for MetS (Table 2).

Similar results were obtained from diabetic women, in which PWNC (OR: 1.009, 95% CI: 1.005–1.012), systolic blood pressure (OR: 1.066, 95% CI: 1.019–1.115), SHH (OR: 22.73, 95% CI: 5.025–99.99), HDL-C (OR: 0.004, 95% CI: 0.000–0.043) (all *P* < 0.01), and TGs (OR: 3.046, 95% CI: 1.047–8.857) (*P* < 0.05) were independent risk factors for MetS. Among anthropometric indices, PWNC was the only independent risk factor; however, other traditional anthropometric parameters did not enter the equation (Table 2).

### ROC curve of anthropometric parameters for MetS

**Fig. 1:**
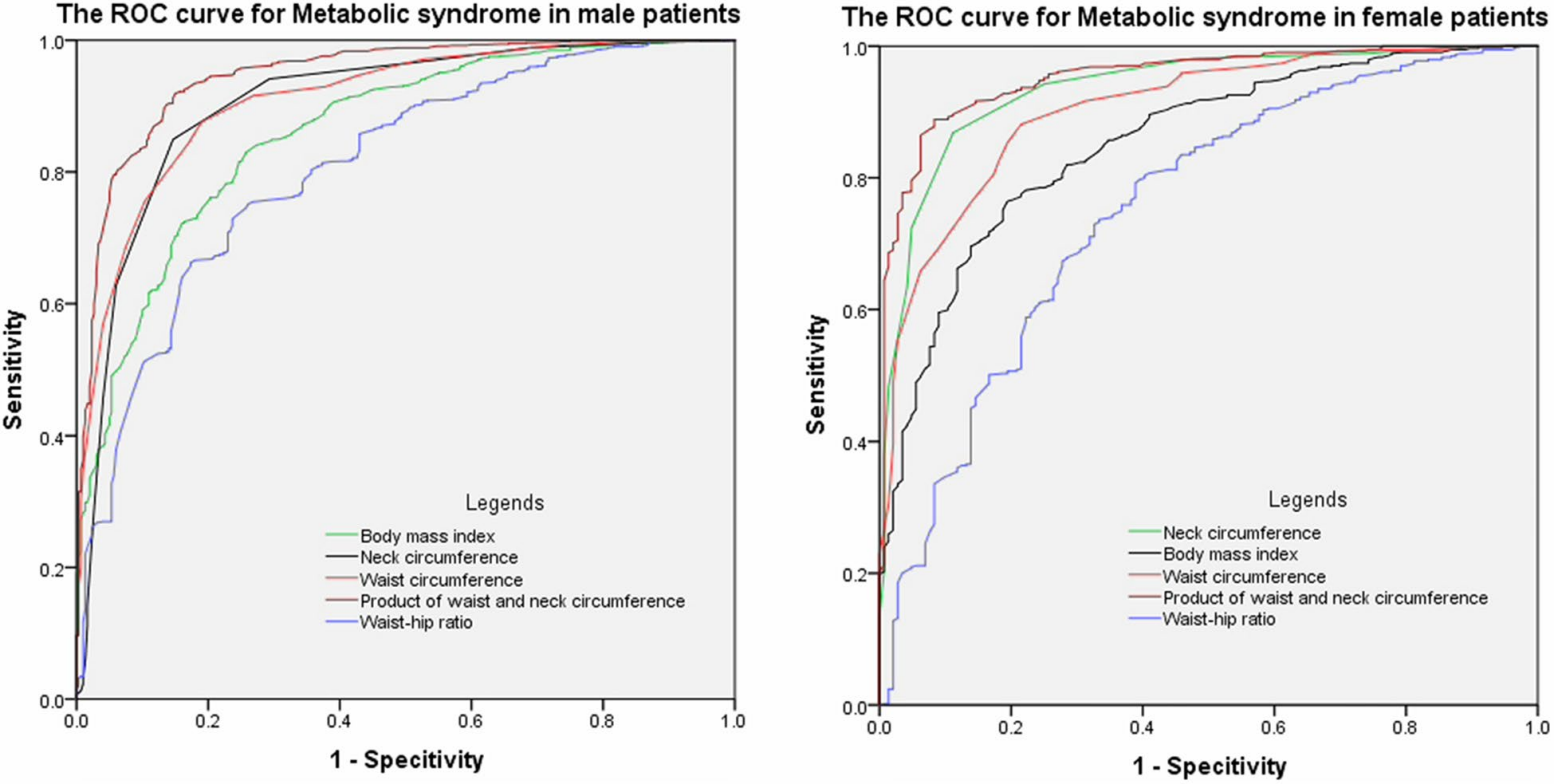
ROC curves of anthropometric parameters as indicators of metabolic syndrome. Left (men), right (women). The area under the ROC curve for the product of waist and neck circumference was larger than that for waist circumference in men and women (DeLong test, *P* < 0.01). Moreover, the area for waist circumference was greater than the body mass index and waist-to-hip ratio in men and women (DeLong test, both *P* < 0.01). Therefore, the product of waist and neck circumference outperformed traditional anthropometric indicators for identifying MetS in both sexes

PWNC, WC, NC, WHR, and BMI displayed significant values in the ROC curve (Fig. 1) for MetS in both sexes (all *P* < 0.01). In male subjects with T2DM, the area under the ROC curve for PWNC, WC, NC, WHR, and BMI was 0.948, 0.913, 0.905, 0.811, and 0.862, respectively (Table 3). The area under the ROC curve for PWNC was larger than that for WC (DeLong test, *P* < 0.01). Moreover, the area for WC was greater than that for BMI and WHR (DeLong test, both *P* < 0.01). Therefore, PWNC was superior to traditional anthropometric parameters in indicating the presence of MetS. The optimal cut-off levels of PWNC, WC, NC, WHR, and BMI that gave the highest sensitivity and specificity were 3542 cm^2^, 90.5 cm, 39.5 cm, 0.959, and 24.8 kg/m^2^ respectively (Table 3).

**Table 3:** ROC curve of different anthropometric parameters for MetS in men

Anthropometric parameters	Area under curve	95% confidence interval	Optimal cut-off level	Sensitivity	Specificity	Youden's index
PWNC**	0.948	0.934–0.962	3542 cm^2^	0.919	0.852	0.771
WC	0.913	0.895–0.931	90.5 cm	0.876	0.818	0.694
NC	0.905	0.884–0.927	39.5 cm	0.849	0.855	0.704
WHR**	0.811	0.784–0.838	0.959	0.728	0.764	0.492
BMI**	0.862	0.839–0.885	24.8 kg/m^2^	0.761	0.798	0.559

*ROC* Receiver operating characteristic curve, *MetS* Metabolic syndrome, *PWNC* Product of waist circumference and neck circumference, *WC* Waist circumference, *NC* Neck circumference, *WHR* Waist-hip ratio, *BMI* Body mass index. The area under the curves compared with WC: ** *P* < 0.01 (DeLong test)

Similar results were obtained from analyses in the female participants; the ROC areas for PWNC, WC, NC, WHR, and BMI were 0.955, 0.905, 0.937, 0.757, and 0.852, respectively (Table 4). PWNC was a better indicator than WC (DeLong test, *P* < 0.01), and WC was better than WHR and BMI (*P* < 0.05) for the presence of MetS in the ROC curve (Fig. 1). The optimal cut-off levels of PWNC, WC, NC, WHR, and BMI that gave the highest sensitivity and specificity were 3130 cm^2^, 85.5 cm, 35.3 cm, 0.925, and 24.5 kg/m^2^ respectively (Table 4). Therefore, PWNC outperformed traditional anthropometric indicators for identifying MetS in both sexes with type 2 diabetes.

**Table 4:** ROC curve of different anthropometric parameters for MetS in women

Anthropometric parameters	Area under curve	95% confidence interval	Optimal cut-off level	Sensitivity	Specificity	Youden's index
PWNC**	0.955	0.938–0.972	3130 cm^2^	0.868	0.937	0.805
WC	0.905	0.879–0.931	85.5 cm	0.882	0.785	0.667
NC	0.937	0.916–0.959	35.3 cm	0.871	0.889	0.760
WHR**	0.757	0.711–0.803	0.925	0.802	0.604	0.406
BMI*	0.852	0.819–0.886	24.5 kg/m^2^	0.694	0.761	0.555

*ROC* Receiver operating characteristic curve, *MetS* Metabolic syndrome, *PWNC* Product of waist circumference and neck circumference, *WC* Waist circumference, *NC* Neck circumference, *WHR* Waist-hip ratio, *BMI* Body mass index. The area under the curve compared with WC: * *P* < 0.05; ** *P* < 0.01 (DeLong test)

## Discussion

To the best of our knowledge, this is the first study to investigate PWNC, a novel anthropometric index, as an obesity indicator for MetS. PWNC was an independent risk factor for MetS in both male and female subjects by logistic regression analysis in our study. It outperformed traditional anthropometric indicators for identifying MetS with type 2 diabetes, and the optimal cut-off value for PWNC was 3542 cm^2^ (sensitivity 0.919, specificity 0.852, and Youden index 0.771) for men and 3130 cm^2^ (sensitivity 0.868, specificity 0.937, and Youden index 0.805) for women in our ROC curve for MetS.

It was estimated that 20–25% of the world’s adult population suffered from MetS [15], and the prevalence of MetS was even higher in diabetic patients; 78.1% of Chinese subjects (77.7% in men and 78.9% in women) with T2DM had MetS in our study. They had more cardiometabolic risk factors than non-MetS patients and were more prone to further cardio- and cerebrovascular disease [3]. The interrelated cardiometabolic risk factors were dyslipidaemia, hyperglycaemia, insulin resistance, hypertension, and mainly obesity.

In the present study, plasma TG levels were higher while HDL-C levels were lower in the MetS group than in the non-MetS group, and high TG and low HDL-C levels were independent risk factors for MetS in the logistic regression analysis. Plasma FPG, FINS levels, and HOMA-IR were all higher in MetS, but only HOMA-IR, instead of FINS and FPG, was an independent risk factor for MetS; thus, low HDL-C, high TGs and IR were essential components of MetS in T2DM patients. The WHO defined the first criteria of MetS and emphasized IR as the major underlying risk factor [5]. IR-mediated increases in circulating free fatty acids play a pivotal role in the development of IR and MetS [16]; IR causes very-low-density lipoprotein overproduction, resulting in hypertriglyceridemia and lower HDL-C concentrations [17]. The relationship between IR and dyslipidaemia might be reciprocal and mutually reinforced. Adipose tissue stores excess energy in the form of TGs; it increases circulating free fatty acids whose delivery to the liver further increases TG synthesis and exacerbates IR [18]. IR, dyslipidaemia, and obesity were at the core of most cases of MetS. Age and diabetes duration were not risk factors for MetS; there were no differences between the two groups in our study. However, we found that MetS group subjects had higher systolic and diastolic blood pressure than the non-MetS group, and hypertension and hypotensors users were independent risk factors for MetS in the logistic regression analysis. These were in accordance with previous findings [4–10]; all MetS definitions included a measure of blood pressure, triglycerides, HDL-C, and fasting glucose; they differed with respect to the selection of obesity measurements.

BMI and WHR were used in the first formalized MetS definition proposed by the WHO in 1998 [5]. Although BMI was a key component of choice to provide a standardized definition of obesity for national surveillance and international comparisons, it did not reflect fat distribution; approximately two-thirds of Chinese adults with obesity would be missed if screening by BMI alone [19]. WHR reflects fat distribution but not total body fat, and obese and lean individuals might have equal WHR values. In the present study, BMI, WHR, and WC were higher in the MetS group than in the non-MetS group, and all displayed significant values in the ROC curve for MetS, but they worked differently. The area under the ROC curve of BMI was 0.862 for males and 0.852 for females; of WHR: males 0.811, females 0.757; and of WC: males 0.913, females 0.905. DeLong tests showed that both BMI and WHR were inferior to WC in indicating the presence of MetS in men and women. Therefore, WC was the best anthropometric indicator for MetS among the three traditional parameters, and WC replaced BMI and WHR as one of the recent diagnostic criteria of MetS [9, 10]. The cut-off value of WC was suggested to be population- and country-specific in different ethnic groups by the IDF definition [7, 10]. The recommended WC threshold for abdominal obesity in Asian adults was ≥ 90 cm in men and ≥ 80 cm in women according to the expert consultation for the WHO proposed in 2004 [20] and used in the MetS criteria [6, 7]. In the present study, the optimal cut-off point of WC was 90.5 cm in men and 85.5 cm in women; the value was similar in men, and it was higher in women than that of WHO criteria, but our results were similar to the latest proposed Chinese Guideline, which defined abdominal obesity as a WC ≥ 90.0 cm for men or a WC ≥ 85.0 cm for women in 2016 [21]. This outcome might be due to the increase in the average WC value in the last decade. Multicentre large-scale studies are required to reach more reliable cut-off points for different ethnic groups, particularly women.

The ACE definition considered both WC and BMI as indicators of obesity in diagnosing MetS [8]. WC is a common and simple surrogate for estimating visceral adipose tissue, while BMI is better for subcutaneous adipose tissue [22], but BMI cannot distinguish between upper-body and lower-body fat. As subcutaneous fat deposited on the lower body had a protective effect against MetS [23] and would eliminate a certain level of pathologic effect of the upper-body fat, the diagnostic value of BMI was inferior to WC in the ROC curve for MetS in our study. However, NC, an indicator of upper-body subcutaneous fat not influenced by low-body fat, could detect MetS among different age groups in China [10]. In the present study, NC was comparable to WC in the ROC curve for diagnosing MetS. The optimal cut-off level of NC was 39.5 cm for men and 35.3 cm for women, indicating the presence of MetS, and our results were similar to those of other studies in the Chinese population [11–13]. In our logistic regression analysis, NC and traditional anthropometric parameters were not independent risk factors for MetS, and only PWNC was identified as an independent risk factor in women, men, and the whole group for MetS. Moreover, PWNC produced the greatest area in the ROC curve among the different anthropometric indices; the area under the curve was 0.948 in men and 0.955 in women, which was larger than that of traditional anthropometric parameters by the DeLong test. PWNC performed better than the most commonly used WC in diagnosing MetS, with the Youden index increasing from 0.694 (that of WC) to 0.771 (that of PWNC) in men, and from 0.667 (that of WC) to 0.805 (that of PWNC) in women. It might be superior to WC and other traditional anthropometric parameters as a novel indicator for diagnosing MetS. NC reflected upper-body subcutaneous fat located in a separate compartment and accounted for cardiometabolic risk [23], while WC was a surrogate of visceral abdominal fat and a generally accepted cardiometabolic risk factor. PWNC, a novel index taking WC and NC together, considering both abdominal fat and subcutaneous fat in the upper body, would provide more information on trunk obesity and confer more cardiometabolic risk information not accounted for by traditional parameters. It might be a new tool for identifying patients with MetS in Chinese diabetic adults.

Our study had two main limitations. First, MetS was not a discrete entity known to be caused by a single factor. Obesity, hyperglycaemia, dyslipidaemia, and hypertension are complicated interrelated components of MetS; the diagnostic value of anthropometric parameters for MetS could be affected by other cardiometabolic risk factors. Second, the recommended anthropometric cut-offs should be racial- and ethnic-specific. Our results from a single centre of Chinese subjects could not be generalized. The diagnostic value of PWNC and its cut-off point for MetS deserves further investigation among different populations.

## Conclusions

PWNC, a novel proposed index combining WC and NC, was superior to traditional anthropometric indices (WC, BMI, and WHR) for the presence of MetS in both male and female adults with T2DM. Further investigations on different individuals (different racial and ethnic groups) are needed to justify it to be one of the diagnostic criteria for MetS definition.

## Data Availability

The data are available from the corresponding author upon reasonable request.

## Abbreviations

MetS: Metabolic syndrome
T2DM: Type 2 diabetes mellitus
CVD: Cardiovascular disease
BMI: Body mass index
WC: Waist circumference
WHR: Waist-hip ratio
NC: Neck circumference
PWNC: Product of waist circumference and neck circumference
SBP: Systolic blood pressure
DBP: Diastolic blood pressure
FPG: Fasting plasma glucose
IR: Insulin resistance
HOMA-IR: Homeostasis model assessment of insulin resistance
HDL-C: High density lipoprotein cholesterol
LDL-C: Low density lipoprotein cholesterol.
TC: Total cholesterol
TG: Triglyceride
ROC: Receiver-operating characteristic curve
OR: Odds ratio
SHH: Subjects with hypertension and hypotensors
